# Micro Electromechanical Systems (MEMS) Based Microfluidic Devices for Biomedical Applications

**DOI:** 10.3390/ijms12063648

**Published:** 2011-06-07

**Authors:** Muhammad Waseem Ashraf, Shahzadi Tayyaba, Nitin Afzulpurkar

**Affiliations:** School of Engineering and Technology, Asian Institute of Technology (AIT), Bangkok 12120, Thailand; E-Mails: Shahzadi.Tayyaba@ait.ac.th (S.T.); nitin@ait.ac.th (N.A.)

**Keywords:** drug delivery system, microfluidics, micropumps, microneedles

## Abstract

Micro Electromechanical Systems (MEMS) based microfluidic devices have gained popularity in biomedicine field over the last few years. In this paper, a comprehensive overview of microfluidic devices such as micropumps and microneedles has been presented for biomedical applications. The aim of this paper is to present the major features and issues related to micropumps and microneedles, e.g., working principles, actuation methods, fabrication techniques, construction, performance parameters, failure analysis, testing, safety issues, applications, commercialization issues and future prospects. Based on the actuation mechanisms, the micropumps are classified into two main types, *i.e.*, mechanical and non-mechanical micropumps. Microneedles can be categorized according to their structure, fabrication process, material, overall shape, tip shape, size, array density and application. The presented literature review on micropumps and microneedles will provide comprehensive information for researchers working on design and development of microfluidic devices for biomedical applications.

## 1. Introduction

**Figure 1:**
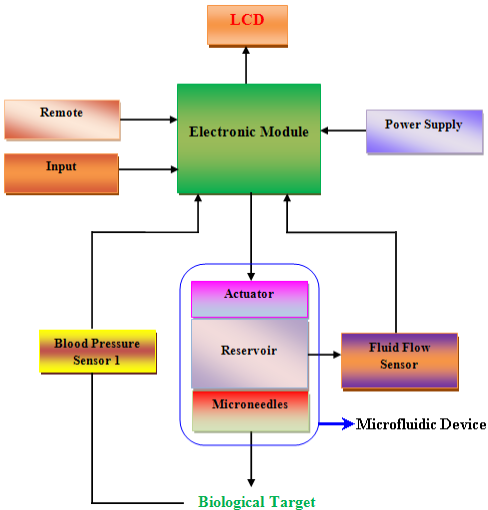
Schematic illustration of transdermal drug delivery (TDD) system.

Microfluidics is a relatively new branch of science and technology which has made extensive progress in the last few years. Microfluidic systems deal with the fluid flow in diminutive amounts, typically a few microlitres (μL) in a miniaturized system. The main functions performed by these systems are sample preparation, purification, separation, reaction, transport, immobilization, labeling, biosensing and detection. Fluid behavior at macro scale is quite different from micro and nano scale. Factors such as surface tension may become dominant in microfluidic devices. When the size of biological samples is close to the flow channels or needles through which the samples are transported, then the sample flow may not be envisaged on the basis of conventional fluidic systems. Considerable research has been made in recent years in the field of microfluidic components, devices, systems and fabrication methods. The use of micro and nano electromechanical systems (MEMS and NEMS) technology has been increasing rapidly to fabricate microfluidic devices for biomedical applications. Due to MEMS and NEMS technology, the fabrication of miniature size and high performance medical devices has become practicable to congregate the critical medical requirements like controlled delivery with negligible side effects, improved bioavailability and therapeutic effectiveness [1,2]. In recent years, the most important advancement of MEMS and NEMS in biomedicine is microfluidic transdermal drug delivery (TDD) systems [3]. TDD systems deal with the movement of pharmaceutical compound through the skin to reach the systemic circulation for subsequent distribution in the human body [4]. TDD system consists of micropumps, microneedles, reservoir, micro-flow sensor, blood pressure sensor, and required electronic circuit for necessary operations. Among them, micropumps and microneedles are the most important components of microfluidic system particularly for drug delivery applications. Micropumps are used for delivery and treatment purposes. Microneedles can be used as stand-alone devices and part of complicated microfluidic system in which microneedles are integrated with other devices in the system. The schematic illustration of transdermal drug delivery system is shown in Figure 1.

In recent years, a few TDD products have been reported and approved by the US FDA. IONSYS (Fentanyl ionophoretic), a product by Alza Corporation was approved in 2006 for patient controlled pain management. Emsam, a product by Bristol-Myers Squibb (Princeton, NJ, USA) was approved in 2006 for major depressive disorder. Fentanyl generic by Watson Pharmaceuticals was approved in 2007 as an analgesic. Neupro, by Schwarz Pharma (Mequon, WI, USA) was approved in 2007 for Parkinson’s disease. Exelon, by Novartis (East Hannover, NJ, USA) was approved in 2007 for dementia [5]. Similarly various researchers have presented microfluidic devices for different medical applications. Particularly micropumps and microneedles have been extensively studied in this decade for biomedicine. But there is still a need to present the latest updates on the development of micropumps and microneedles for biomedicine because these devices are still at the research level and have limited availability for commercial use. Some earlier reviews on various applications of MEMS in the biomedical field have been reported, such as the therapeutic microsystem, surgical microsystem and drug therapy. These reviews provide basic information on various devices such as microneedles, micropumps, micro-reservoirs, *etc*. [6–9]. Various researchers have reported reviews on design and development of micropumps only [10–14]. Laser and Santiago [10] presented a comprehensive review on micropumps. But the review did not cover some actuation methods, e.g., ion conductive polymer film (ICPF), development of evaporation micropump and advance applications of micropumps in biomedicine. Woias [11] presented a concise overview of different types of micropumps and their applications. However, the electrowetting micropump, evaporation micropump and ICPF have not been described in the review. Tsai and Sue [12] reported introductory overview on the importance of micropumps for medical applications, but significant details about the applications of various kinds of micropumps for drug delivery have not been presented. Nisar *et al*. [13] presented a comprehensive and good review on various types of micropumps and their applications in biomedical applications. Some key features of micropumps like actuation techniques, performance parameters, working principles, structure, fabrication and applications have been reported, but this review has not covered latest developments in micropumps for biomedical applications. The review does not provide up-to-date information about bio-MEMS devices as there is an exponential increase in design and development in the bio-medicine field. Amirouche *et al*. [14] presented a review on current developments in micropumps. The focus of this review was on mechanical micropumps and their applications in the biomedical field. However this review has not covered non-mechanical type of micropumps. Grayson *et al*. [15] reported a brief review on various integrated MEMS devices such as biosensors, stents, immunoisolation devices, reservoirs, microneedles, *etc*. This review has not described all parameters of MEMS devices like design, development, actuation methods, fabrication techniques, *etc*. Karman *et al*. [16] reported a very basic and introductory review on drug delivery devices like micropumps, microneedles, microvalves, microactuators, microreservoirs, *etc*. This review has not covered important parameters such as actuation techniques, working principles, performance constraints, design, fabrication and applications of MEMS devices. Bao-jian *et al*. [17] presented information on the development and applications of MEMS based microneedles. This review has not covered some important aspects of design and development, forces experienced by microneedles, testing, structural/fluidic analyses, *etc*. Khanna *et al*. [18] reported a review on the particular design requirements of microneedles for diabetic therapy. This review has not covered the key parameters like development, fabrication, failure analysis, *etc*. Sachdeva and Banga [19] reported good comprehensive review on microneedles design, development, safety and regulatory issue, therapeutic applications and limitations of microneedles for commercialization. However, this review has not described the fabrication techniques of microneedles, failure of microneedles due to various applied forces, structural and fluidic analysis and integration issues of microneedles with micropumps. All reviews that have been discussed above present the information about micropumps or microneedles only. Here the authors have presented a review on micropumps and microneedles that covers most recent advancement of MEMS technology in biomedicine. This is the first comprehensive and updated review that covers latest information of microfluidic devices regarding the design, development, actuation methods, performance parameters, working principles, structure, fabrication techniques, material used for fabrication, safety issue, challenges, limitations of commercialization and applications. This comprehensive review will be helpful for researchers who would like to work in the fast growing field of bio-MEMS and bio-NEMS.

## 2. Micropumps

Pioneering work on micropumps started in the 1970s and developments based on microfabrication technology was initiated in the 1980s. The MEMS based micropump was developed in 1990s. The micropump is the main component of drug delivery system that provides the actuation mechanism to deliver specific volumes of therapeutic agents/drugs from the reservoir. The requirements for drug delivery include a minimum flow rate in order of 10 μL per minute or more, small size and high reliability [13]. Normally a micropump consists of the following components: diaphragm membrane, chamber, actuator, microchannels, microvalves, inlet, outlet, *etc*. Micropumps can be categorized into two classes: One type has a mechanical moving part and is known as a mechanical micropump; the other has no moving part and is known as a non-mechanical micropump.

### 2.1. Design Specifications and Parameters of Micropumps

Design of micropumps plays an important role for practical applications of devices. To develop a suitable design of micropumps for real time applications, it is very important to understand terms like actuator, valves, chamber or reservoir, nozzle diffuser mechanism and pumping parameters properly.

#### 2.1.1. Actuator

The actuator is the necessary and driving part of a micropump that converts energy into motion. It is used to provide force for fluid flow in micropumps. The actuator takes energy from electricity, heat, liquid pressure, air pressure and converts it into some kind of motion. In most micropumps reported in literature, the actuation disk is attached with membrane which is used to push the fluid. Some types of time diaphragm are fabricated in such a way that it produces energy itself which pushes the fluid. In peristaltic micropumps more than one actuator is fabricated sequentially.

#### 2.1.2. Valves

In micropumps, valves are used to control the fluid flow by opening, closing and partially hindering passageways. In microfluidic systems, active and passive valves have been reported. In passive valves there is no actuation mechanism. The control of fluid flow is dependent on the pressure difference in liquid chamber and the fluid flow is normally in one direction. In active valves, active elements are present for opening and closing that are operated by an external actuation source. Mostly, separate components have been reported for active micro-valves for regulating the fluid flow in microfluidic systems. It is very easy to control the active valves but they are more complicated in integrated microfluidic system.

#### 2.1.3. Chamber or Reservoir

Chamber design is very critical in microfluidic systems and it can significantly influence the volume stroke, pressure characteristics and nozzle-diffuser loss coefficients. Most of the micropumps reported in literature have a single chamber configuration. But in order to improve the performance, two or three chamber micropumps have also been reported. Micropumps in which pumping chambers are arranged sequentially or fabricated in such a way that the multiple chambers are in series or in parallel arrangements, are known as peristaltic micropumps.

#### 2.1.4. Nozzle/Diffuser Element

**Figure 2:**
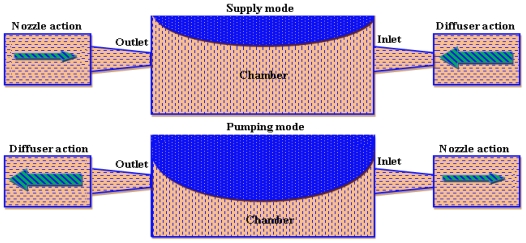
Schematic of nozzle/diffuser element.

Nozzle/diffuser element is mostly used in valveless micropumps as a flow rectifier. A schematic illustration of the nozzle/diffuser action in micropumps is shown in Figure 2. Nozzle/diffuser element works in such a way that during supply mode more fluid enters in the chamber through an inlet than fluid that exiting the outlet. While in pump mode the reverse action occurs. Stemme and Stemme [20] were the first to report valveless miniature micropumps in which they used a nozzle/diffuser element as flow rectifying element.

#### 2.1.5. Pumping Parameters

Various design parameters are important to optimize the performance of micropumps such as maximum flow rate (Q_max_), pump power (P_pump_), maximum back pressure (h_max_) and pump efficiency (η). Q_max_ is highest at zero h_max_ and Q_max_ is zero when highest value of h_max_. For incompressible flow, the pump head (h) can be calculated from the steady flow energy equation [21].

(1) $$h={\left(\frac{P}{\gamma }+\frac{u^{2}}{2g}+Z\right)}_{out}-{\left(\frac{P}{\gamma }+\frac{u^{2}}{2g}+Z\right)}_{in}$$

Where, *P* is pressure, *γ* is pressure head, $\frac{u^{2}}{2g}$ is velocity head and *Z* is elevation.

The pump efficiency (η) in the form of power can be expressed as:

(2) $$\eta =\frac{P_{pump}}{P_{actuator}}$$

Ideally, losses are zero and both quantities *P_pump_* and *P_actuator_* are identical. Efficiency is governed by frictional losses, fluid leakage losses and losses due to imperfect pump construction. The total efficiency can be expressed as [21].

(3) $$\eta =\eta_{m}\eta_{v}\eta_{h}$$

Where *η_m_*, is mechanical efficiency, *η_v_* is volumetric efficiency and *η_h_* is hydraulic efficiency.

### 2.2. Mechanical Micropumps

The mechanical micropumps have moving parts so require a physical actuator for the pumping process. The most common mechanical micropumps are displacement type micropumps that involve a pumping chamber which is closed with a flexible diaphragm. The fluid flow is achieved by the oscillation of a diaphragm. Due to these oscillations, the pressure (ΔP) is created. This pressure is a function of stroke volume (Δ*V*) inside the chamber produced by the actuator. The actuator has to run itself with the dead (*V*_0_) volume in chamber. Compression ratio is the important parameter for mechanical diaphragm type micropumps. The compression ratio is defined by the equation (4):

(4) $$ɛ=\frac{\Delta V}{V_{0}}$$

The performance of mechanical micropump is normally limited by its mechanical components. The piezoelectric, electrostatic, thermopneumatic, electromagnetic, bimetallic, ion conductive polymer films (ICPF), phase change and shape memory alloy (SMA) are examples of mechanical micropumps. A detailed description of mechanical micropumps is given below.

#### 2.2.1. Piezoelectric Micropumps

The conversion of mechanical energy to electronic signal (voltage) and *vice versa* is known as the piezoelectric effect. The materials which exhibit piezoelectric effect normally have no center of symmetry in their structure. A stress applied to such materials will alter the separation between the positive and negative charges that leads to the net polarization at the surface. An electrical field with voltage potential is created in those materials due to the polarization. This property can be used to form the actuator, micropump, inkjet printer head, *etc*. The effectiveness of energy and *vice versa* can be expressed by factor *K*:

(5) $$K^{2}=\frac{Out\;put\;of\;mechnical\;energy}{Input\;of\;eletrical\;energy}$$

**Figure 3:**
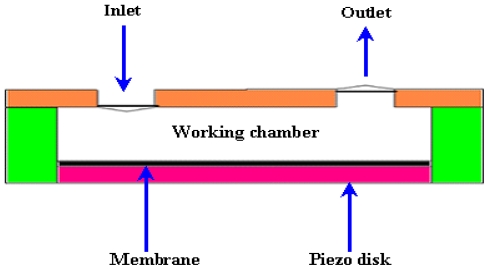
Piezoelectric micropump.

Piezoelectric actuator shows large actuation and fast response time, but the fabrication of such materials is complicated on a single chip. Piezoelectric micropumps exhibit small stroke volume at high voltages. A schematic of a piezoelectric micropump is shown in Figure 3.

The first piezoelectric micropump was fabricated using micromachining technology by Van Lintel *et al*. [22]. The micropump consisted of a pumping chamber, passive silicon (Si) check valve, and a thin glass membrane actuated by piezo disk. The maximum flow rate of 8 μL/min and back pressure of 9.8 kPa were observed at applied 125 V with 1 Hz frequency. Esashi *et al*. [23] reported a three layers piezoelectric pump with flow rate of 15 μL/min and back pressure of 6.4 kPa at applied 90 V with 30 Hz frequency. Olsson *et al*. [24] reported a two chamber piezoelectric micropump to improve the performance. Koch *et al*. [25] presented piezoelectric micropump based on screen printing of PZT (Lead Zirconate Titanate) on Si membrane. The flow rate of 120 μL/min and back pressure 2 kPa were observed at applied 600 V with 200 Hz frequency. Schabmueller *et al*. [26] fabricated piezoelectric micropump with passive valves. The flow rate of 1500 μL/min and back pressure of 1 kPa were achieved using ethanol. Feng and Kim [27] reported piezoelectric micropump that consisted of one way parylene valves. The flow rate of 3.2 μL/min and back pressure of 0.2 kPa were observed at applied 80 V with lower power consumption of 3mW. Geipel *et al*. [28] reported a novel design of micropump with back flow pressure independent flow rate. The back pressure independency was reported up to 20 kPa at low frequency. Trenkle *et al*. [29] reported a piezostack actuated peristaltic micropump. The flow rate of 40 μL/min was obtained at the frequency of 28.6 Hz using water. The flow rates were observed to be independent of backpressure up to 7 kPa, with a maximum backpressure of 45 kPa at 140 V. Johari *et al*. [30] reported the fabrication of a piezoelectric micropump for drug delivery system using two optical masks. Fluidic characteristics analysis was performed using CoventorWare simulator. Wang *et al*. [31] studied the effect of longitudinal flow asymmetry on pumping capability by using a simple pumping system comprised of a piezoelectric buzzer imbedded in a channel. Ali *et al*. [32] studied the dynamic piezoelectric micropump process. The quantitative measurement of the pressure generated, applied electrical field, frequency and length of the actuator, were observed. Liu *et al*. [33] proposed a disposable high performance piezoelectric micropump with four chambers in serial connection for closed loop insulin therapy system. Outflow resolution of 6.23 × 10^−5^ mL/pulse was observed. The maximum backpressure of 22 kPa was reported at applied voltage of 36 V_pp_ and 200 Hz frequency.

#### 2.2.2. Electrostatic Micropumps

Electrostatic micropumps involve electrostatic forces for actuation mechanism. Electrostatic force F is defined as “the electrical force of attraction and repulsion induced by an electric field (E)”. The like charges repel each other and unlike charges attract each others. The electrostatic force applied on the electrostatic plates can be expressed by the equation (6):

(6) $$F=\frac{dW}{dX}=\frac{1}{2}(ɛA\frac{V^{2}}{X^{2}})$$

Where, *F* is electrostatic attraction force, *W* is energy stored, *ɛ* is dielectric constant, *A* is area of electrodes, *X* is electrode spacing and *V* is applied voltage.

**Figure 4:**
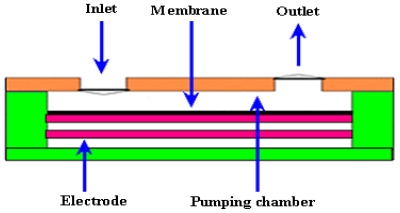
Electrostatic micropump.

Electrostatic actuation is widely used in microfluidic devices. The fabrication of such mechanisms on electronic chip is very easy, but electrostatic actuator has only a small stroke, typically 10 μm. The main advantages of electrostatic micropump are low power consumption and fast time response. The schematic of an electrostatic micropump is shown by Figure 4.

The first electrostatic micropump was fabricated by Judy *et al*. [34] using surface micromachining technology. It consisted of active check valve, chamber and active outlet valve. Pumping results were not reported. The first experimental results of electrostatic micropump were reported by Zengerle *et al*. [35]. The flow rate of 70 μL/min and back pressure of 2.5 kPa were observed at applied 170 V with frequency 25 Hz. Cabuz *et al*. [36] presented dual diaphragm electrostatic micropump using injection molding technique. Micropump was capable of bidirectional operation but only used for gases. The flow rate of 30 μL/min was observed at applied 160 V with frequency of 30 Hz and power of 8 mW. Machauf *et al*. [37] presented membrane based electrostatically actuated micropump across the working fluid. The concept was based on high and low electric permittivity of working fluid. This pump was limited only for conducting fluid. The flow rate of 1 μL/min was achieved at 50 V. Astle *et al*. [38] proposed a pumping mechanism using electrostatic actuation for gas chromatograph applications. The flow rate of 3 mL/min and backpressure of 7 kPa were observed at frequency of 14 kHz. Lee *et al*. [39] fabricated and tested a peristaltic electrostatic gas micropump that employed fluidic resonance for high flow rate and multi stage peristaltic configuration. The micropump presented the pressure ranges from 7.3 to 3.3 kPa and flow rates from 0.29 to 0.07 sccm at the duration time ranges from 0.05 and 0.35 cycles for opening of valves. Liu [40] reported the “pull in phenomena” in electrostatic micropump using reduced order model of membrane. Various parameters like radius, thickness, initial gap, residual stress on pull in voltage and pull in position were investigated. Lil *et al*. [41] presented the modeling of micropump membrane with electrostatic actuator. MATLAB platform was used for modeling. The resonant frequency of 635 Hz for silicon electrostatic actuating membrane was calculated. Using FEM, 680 Hz frequency was reported.

#### 2.2.3. Thermopneuamtic Micropumps

In thermopneumatic micropumps, the actuator is based on thermal expansion. The chamber is full of air and thermopneumatic micropump is expanded and compressed periodically by the heater and cooler. The periodic change in volume of chamber provides the membrane with a regular momentum that results in fluid out flow. The pressure increase is expressed by the equation (7).

(7) $$\Delta P=E\;(\beta \Delta T-\frac{\Delta V}{V})$$

Where, Δ*P* is pressure change, Δ*T* is temperature change, *β* is thermal expansion, $\frac{\Delta V}{V}$ is a percentage of volume change.

**Figure 5:**
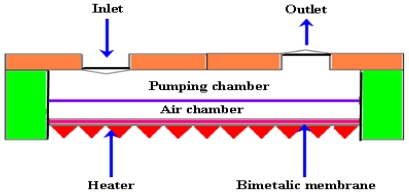
Thermopneumatic micropump.

The thermopneumatic type of micropump generates relatively strong pressure and displacement of membrane. However, the driving power has to be constantly maintained above a certain level. The schematic diagram of thermopneumatic micropump is shown by the Figure 5.

The first thermopneumatic micropump based on microfabrication was proposed by Van De Pol *et al*. [42]. The flow rate of 34 μL/min was observed at applied voltage of 6 V with temperature around 30 ˚C. Jeong and Yang [43] reported a thermopneumatic micropump with corrugated diaphragm. The flow rate of 14 μL/min was observed at applied voltage of 8 V with frequency of 4 Hz. A thermopneumatic micropump consisting of a thin film heater, flow strictor and two reservoirs has been proposed by Cooney and Towe [44]. The maximum flow rate of 1.4 μL/min for 4.5 h was observed with an average power of 200 mW. Kim *et al*. [45] proposed a thermopneumatic micropump with a glass layer, indium tin oxide heater, polydimethylsiloxane (PDMS) chamber, PDMS membrane and PDMS cavity. The flow rate of 0.078 μL/min was achieved at applied voltage of 55 V with frequency of 6 Hz. Jeong and Konishi [46] fabricated a peristaltic micropump consisting of three cascaded thermopneumatic actuators and microfluidic channel connecting two fluidic inlet/outlet ports. The flow rate of 73.9 μL/min was achieved for the de-ionized (DI) water at zero backpressure. Chia *et al*. [47] proposed a novel thermopneumatic peristaltic micropump comprised of two separate zones for air heating and fluid squeezing. The temperature elevation of 2.0 K was reported on the fluid pumping area. Tan *et al*. [48] fabricated a peristaltic micropump by bonding a PDMS part with microchannels to the PDMS/PMMA (polymethylmethacrylate) part where PDMS/adhesive membrane worked like a pneumatic actuator. The maximum flow rate of 96l μL/min was achieved.

#### 2.2.4. Electromagnetic Micropumps

Electromagnet is a kind of magnet that is based on the combination of electric and magnetic fields. When the current passes through the coils the magnetic field is produced. The strength of electromagnet can be easily varied by changing the electric current flowing through the coils. The force experienced by the point charge due to the electromagnetic field is known as the Lorentz force. The Lorentz force can be expressed by equation (8).

(8) F=I(I×B)

Where, *F* is force and *B* is magnetic field.

**Figure 6:**
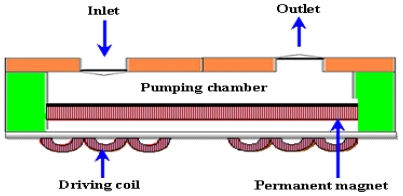
Electromagnetic micropump.

Electromagnetic actuation is large and covers a longer distance as compared to electrostatic actuation. It needs low voltage but an external source is required for actuation such as a permanent magnet. On small scale, this type of actuation has no benefit because it is reduced by the cube of scaling factor. The driving coils or permanent magnets bond directly with the membrane and provide a magnetic field. However, at the same time, the size is compromised. Usually electromagnetic micropumps have high power consumption and heat dissipation. A schematic of an electromagnetic micropump is shown in Figure 6.

The first electromagnetic micropump with 7 μm thick Ni_80_Fe_20_ film electroplated on 17 μm thick Si membrane was proposed by Zheng and Ahn [49]. The maximum flow rate of 20 μL/min was observed at applied voltage of 3 V with 5 Hz frequency and 300 mA induced current. A plastic micropump with electromagnetic actuation has been reported by Bohm *et al*. [50] that consisted of two folded valves with a thin membrane in center, inlet/outlet at bottom and pump membrane at top. The maximum flow rates of 40,000 μL/min for air and 2100 μL/min for water were observed with power consumption of 0.5 W. A four layer electromagnetic micropump was designed and its static/dynamic properties were investigated by Gong *et al*. [51]. The membrane deflection by different magnetic driving forces was analyzed by ANSYS FEM. The maximum flow rate of 70 μL/min was observed at frequency of 125 Hz. Yamahata *et al*. [52] reported a PMMA micropump with electromagnetic actuation. The maximum flow rate of 400 μL/min and back pressure of 1.2 kPa were observed at resonant frequencies of 12 Hz and 200 Hz. Su *et al*. [53] reported the analysis and fabrication of a valveless electromagnetic micropump with two parallel flexible diaphragms. The maximum flow rate of 6 μL/s and the displacement of 0.30 mm were observed at 100 Hz frequency with 0.3 A induced current. Balaji *et al*. [54] reported the design, fabrication and testing of a flat pump with millimeter thickness. The maximum flow rate of 15 μL/min was observed at applied voltage of 2.5 V with 68 Hz frequency and 19 mA current. Yu-feng *et al*. [55] reported a parallel dynamic micropump with valve, diaphragm and electromagnetic coil. The maximum flow rate of 6 μL/s and the diaphragm displacement of 30 μm were observed at 100 Hz frequency with 0.3 A of current. Shen *et al*. [56] fabricated and characterized a reciprocating PMMA ball valve micropump with electromagnetic actuation. The micropump showed a backpressure of 35 kPa and flow rate of 6 mL/min at 2 W electromagnetic actuation power with 20 Hz resonant frequency. Halhouli *et al*. [57] worked on the design of a novel electromagnetic pump that based on the rotation of two hard magnets kept in channel, with opposing polarity. The maximum flow rate of 13.7 mL/min at 200 rpm and a pressure of 785 Pa at 136 rpm were observed.

#### 2.2.5. Bimetallic Micropumps

**Figure 7:**
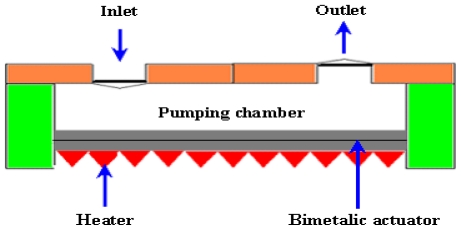
Bimetallic micropump.

Bimetal refers to an object that is composed of two different metals jointed together. The thermal expansion coefficients of these metals are different. The deflection of a diaphragm made of bimetallic materials is induced against thermal alternation as long as the two chosen materials possess adequately discriminative thermal expansion factors. A block diagram of bimetallic micropump is shown in Figure 7.

Zhan *et al*. [58] reported Si based bimetallic micropump with 10 μm thick layer of aluminum (Al) on Si substrate. The flow rate of 45 μL/min and back pressure of 12 kPa were observed at applied voltage of 5.5 V with 0.5 Hz frequency. Zou *et al*. [59] designed a micropump that operated on both bimetallic thermal actuation and thermal pneumatic actuation mechanisms. When the bimetallic actuator made of Al/Si membrane was heated, the membrane deformed in downward direction. At the same time, the gas in the air chamber expended due to the heat to support bimetallic actuation. The flow rate of 336 μL/min was achieved when the open pressure was 0.5 kPa. A novel micropump operated on bimetallic and electrostatic actuation mechanisms was reported by Pang *et al*. [60]. Experimental results showed that the on/off flow ratio of the micropump was 180. Yang *et al*. [61] presented a bimetallic thermally actuated membrane micropump that consisted of two chips, pump chamber, two bimetallic actuators and two check valves. The maximum flow rate of 43 μL/min was achieved at applied voltage of 16 V and 0.9 Hz frequency. The forces generated through bimetallic actuation are large and the implementation is simple. Usually the thermal expansion coefficients of materials that are involved in bimetallic micropumps are small. That is why the diminutive deflections are achieved in bimetallic actuation mechanism. The bimetallic micropumps require low voltage values as compared to other micropump types. But the drawback of bimetallic micropumps is that they are not suitable to work at high frequencies.

#### 2.2.6. Ion Conductive Polymer Film (ICPF) Micropumps

**Figure 8:**
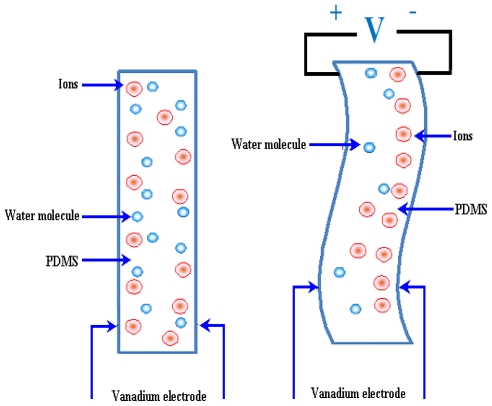
ICPF micropump.

ICPF actuator shows high speed response. However, the positioning control is difficult. The core layer of ICPF is made of a sort of perfluorosulfonic acid polymer. Physically it looks like a “sandwich” diaphragm between two thin films that are placed on both sides of the polymer. These two films have high electrical conductivity. One end of the diaphragm is fixed and the ICPF diaphragm can be controlled by bending in the direction of either upside or downside as long as an appropriate pair of voltages is applied at the electrodes. The ICPF actuator is commonly called an artificial muscle because of the large bending displacement, low actuation voltage and biocompatibility. A schematic of ICPF and the bending principle is shown in Figure 8.

ICPF actuators have been developed for various applications. Guo *et al*. [62] reported a new model of micro catheter with active guide wire that had two bending degrees of freedom using ICPF actuator. Tadokoro *et al*. [63] developed multi-degree-of-freedom (DOF) micro motion devices using ICPF soft gel actuator. Guo and Asaka [64] proposed an underwater fish like microrobot using ICPF actuator as the servo actuator swimming motion with three degrees of freedom. Nguyen *et al*. [65] reported the design and fabrication of a flap valve ionic polymer/metal composite micropump with the diaphragm supported by a flexible material. A maximum flow rate of 760 μL/min and backpressure of 1.5 kPa were observed at the applied voltage of 3 V with 3 Hz frequency. Chen *et al*. [66] proposed the design of an integrated sensory actuator. The polyvinylidene fluoride (PVDF) films were used for simultaneous feedback of bending and force outputs of the actuator. Fang and Tan [67] proposed a control oriented model to envisage the deformation of diaphragm and the flow rate. Experimental results of the polypyrrole (PPy) actuated micropump showed that the maximum flow rate of 1260 μL/min was observed at the voltage of 4 V.

#### 2.2.7. Phase Change Micropumps

**Figure 9:**
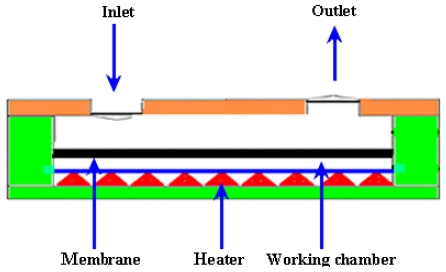
Phase change micropump.

The basic principle used in phase change type actuators and micropumps is the vaporization and condensation phenomenon. In vaporization, the phase transition occurs from liquid phase to vapor phase. While in condensation, the change of the physical state occurs from gaseous phase to liquid phase. The phase change type micropump consists of a heater, diaphragm and working fluid chamber. A schematic of phase change micropump is shown in Figure 9.

Sim *et al*. [68] proposed a phase change type micropump consisting of a pair of Al flap valves and a phase-change type actuator. The actuator comprised of a heater, working fluid chamber and silicone rubber diaphragm. The diaphragm was actuated by the vaporization and the condensation of the working fluid in the chamber of the pump. The maximum flow rate of 6.1 μL/min was achieved at applied voltage of 10 V with 0.5 Hz frequency and 60% duty ratio for zero pressure difference. Boden *et al*. [69] reported a high pressure micropump with polymeric paraffin actuation. The flow rate of 74 μL/min was achieved at a low voltage waveform with water as a pumping fluid. When the pressures up to 1MPa were applied on the valves, the micropump showed no leakage. Sim *et al*. [70] reported the fabrication and testing of a micropump comprised of a pair of Al flap valves and a phase change type actuator. The actuator was composed of a heater, diaphragm and fluid chamber. The maximum flow rate of 97 μL/min was observed at applied voltage of 8 V with 70% duty ratio and 2 Hz frequency for zero pressure difference.

#### 2.2.8. Shape Memory Alloy (SMA) Micropumps

**Figure 10:**
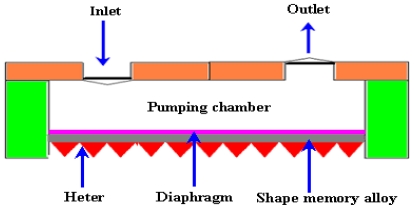
SMA micropump.

SMA are the metals which exhibit two very unique properties such as pseudo elasticity and the shape memory (SM) effect. They have the capability of changing their shapes upon application of an external stimulus. The SM effect involves a phase transformation between two solid phases. At high temperature the phase is called austenite and at low temperature the phase is called martensite. SMA starts in martensite phase and transforms into austenite phase after being heated. This property of materials is useful to make SMA micropumps. A schematic of an SMA micropump is shown in Figure 10.

The first thin film SMA micropump with two different actuation configurations was reported by Benard *et al*. [71]. The pump was driven by an electrical drive signal provided directly through the Titanium/Nickel (Ti/Ni) thin films, resulting in Joule heating induced phase transformation that initiated the SM effect. The maximum flow rate of 50 μL/min was observed at 0.9 Hz frequency. Makino *et al*. [72] reported the development of SMA actuated micropump to use in micro analysis and micro dosage systems. The maximum flow rate of 0.4 μL/cycle was observed at a bias pressure of 100 kPa. Xu *et al*. [73] developed a micro SMA pump composed of a NiTi/Si composite membrane, pump chamber and two inlet/outlet check valves. The flow rate of 340 μL/min and back pressure of 100 kPa were achieved. Shuxiang and Fukuda [74] developed SMA micropump composed of SMA coil actuator, two diffusers, pump chamber and a casing. The maximum flow rate of 500–700 μL/min was achieved by changing the frequency. Zhang and Qiu [75] reported a Ti/Ni/Copper (Cu) shape memory thin film micropump comprised of a TiNiCu/Si driving membrane, pump chamber and two inlet and outlet check valves. The hysteresis width ΔT of 9 °C was observed. Setiawan [76] reported the performance assessment of SMA spring as actuator for gripping manipulation. The SMA actuator was a TiNi tensile spring with diameter of 50 mm wire and 350 gram hanging mass. SMA have many attractive properties like high force to volume ratio, ability to recover large transformation stress and strain upon heating and cooling processes, high damping capacity, chemical resistance and biocompatibility. Usually the deformation of SMA cannot be precisely controlled and investigated due to temperature sensitivity. Additionally, the designs based on TiNi film devices with more practical, effective and complex characteristics, are required through multiple DOF and compact structures. Recently reported mechanical micropumps are listed in Table 1.

### 2.3. Non-Mechanical Micropumps

The non-mechanical micropumps have no moving mechanical part so that generally they need a type of mechanism that can convert non-mechanical energy into kinetic momentum. In general, non-mechanical pumps do not need physical actuation components so the geometry, design and fabrication of these micropumps are relatively simple and easy. These micropumps have certain limitations, such as the use of only low conductivity fluids and the actuation mechanisms interfere with the pumping liquids. A detailed description of non-mechanical micropumps is given below.

#### 2.3.1. Electroosmotic (EO) Micropumps

**Figure 11:**
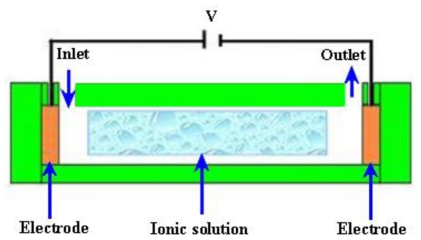
EO micropump.

EO flow is the motion of the liquid that is induced by an applied potential across a capillary tube or microchannels. The fluid with electric conductivity feature is driven by appropriately exerting an external electrical field upon the channel walls that are naturally charged. A schematic diagram of an electroosmotic micropump is shown in Figure 11.

Zeng *et al*. [81] fabricated an EO micropump that used DI water as working fluid. The maximum flow rate of 3.6 μL/min and pressure of 2026.5 kPa were obtained at applied voltage of 2 kV. Takemori *et al*. [82] reported an EO micropump with high pressure. The flow rate of 0.47 μL/min and pressure of 72 kPa were observed at applied 3 kV. Hu and Chao [83] investigated the EO flow in EO micropump with an overlapped electrical double layer (EDL). The results showed that the flow was relatively different from the channel with a dimension greater than the EDL, which demonstrated plug like flow properties. Good *et al*. [84] performed the mathematical modeling and experimental testing of water activated micropump that was actuated using the osmotic effect. The maximum flow rate of 17 μL/min/mg of dry polymer particles, with a 355–425 μm diameter, was achieved. Ryu *et al*. [85] proposed a biodegradable osmotic micropump for long use and controlled discharge of basic fibroblast growth factor (bFGF). The release of bFGF was regulated at a rate of 40 ng/day for duration of four weeks. Yairi and Richter [86] developed an EO micropump based on voltage control. The flow rate of 0.054 mL/min and pressure of 5.5 kPa were achieved. Borowsky *et al*. [87] fabricated a high pressure EO micropump and tested the performance of fluid dynamic. The maximum flow rate of 85 μL/min and pressure of 25 atm were achieved. Wang *et al*. [88] reported the general characteristics, fabrication technologies and applications of EO micropumps. The transport of various solutions compositions into capillaries can cause problems in the flow constancy of an EO pumped system in some applications. Sometimes flow rates are modified due to adsorption of compounds from the samples or sample matrix on the surfaces of the pumping elements. This problem can be solved by separating the pump fluid from the sample and reagent solutions in the analytical system.

#### 2.3.2. Electrowetting (EW) Micropumps

**Figure 12:**
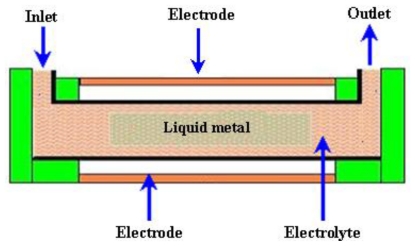
EW micropump.

EW is a microfluidic phenomenon that is currently used as a driving mechanism for fluidic devices. EW involves modifying the natural surface tension or capillary forces intrinsic to an oil and water interface at small length scales. At less than 1 mm distance, the electrical and surface tension forces are much stronger than gravity. The digital EW is applied to control the surface tension between solid phase electrode and liquid phase droplet. A schematic of an EW micropump is shown in Figure 12.

Yun *et al*. [89] reported a continuous EW micropump. For the actuation energy of micropump, the surface tension induced motion of mercury drop in a microchannel filled with electrolyte was used. The micropump consisted of a stack of three wafers bonded together. The flow rate of 70 μL/min and pressure of 0.8 kPa were achieved at 2.3 V with frequency of 25 Hz and power consumption of 170 μW. Hoshino *et al*. [90] reported the pico liter liquid actuation in a microinjector by using a pulled glass tube as the device structure. The tube caused pumping and ejection by EW on dielectrics. 500 picoliter water was pumped up at the maximum applied voltage of 1400 V. In pumping pressure, an increase value of 0.6 Pa was calculated. Colgate and Matosumoto [91] reported a detailed model of a test device showing liquid flow in a small channel for the study of EW. EW gives direct fluid pumping without any moving mechanical parts that can be valuable in many application areas of microelectronic devices. The initial results showed that EW might be used to get pressures on the order of 0.01 MPa in a 10 μm radius channel. Chang *et al*. [92] reported the driving characteristics of the EW-on-dielectric device with aluminum oxide (Al_2_O_3_) deposited by using the method of atomic layer deposition. When the voltage was applied between control electrode and reference electrode then the flow of 2 μL for water droplet in an air environment was achieved.

#### 2.3.3. Electrochemical Micropumps

The most common feature of electrochemical micropumps is the generation of bubbles by electrolysis in which the decomposition of water occurs into its constituents, such as hydrogen gas (H2) and oxygen gas (O2), when the current is passed through water. During this mechanism, the key component is a bubble reservoir filled with a redox electrolyte solution. The reaction of electrolysis can be described by the equations (9) and (10).

At Anode

(9) $$2{\text{H}}_{2}\text{O}\to 4{\text{H}}^{+}+4{\text{e}}^{-}+{\text{O}}_{2}(\text{g})$$

At Cathode

(10) $$2{\text{H}}_{2}\text{O}+2{\text{e}}^{-}\to 2{\text{OH}}^{-}+{\text{H}}_{2}(\text{g})$$

**Figure 13:**
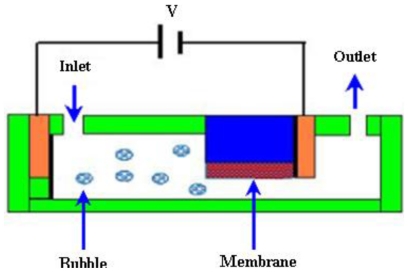
Electrochemical micropump.

A schematic of electrochemical micropump is shown in Figure 13.

Suzuki and Yoneyama [93,94] fabricated an electrochemical syringe pump by using micromachining for low operating voltage and power consumption. A microfluidic system was developed by integrating an on-chip micropump and check valves that worked through a H_2_ bubble generated electrochemically. Thin film electrodes were used with a platinum black working electrode. PDMS substrate was used to make flow channels and containers for electrolyte solutions. Two dye solutions were transported and merged in a flow channel and sheath flows were observed. Yoshimi *et al*. [95] developed an artificial synapse using the electrochemical micropump. The micropump consisted of a glass nozzle and two blackened platinum electrodes filled with a neurotransmitter solution for the electrolysis process. To drive the solution towards the neuron, a potential difference of 3.0 V was applied to the electrodes. Kim *et al*. [96] reported a PPy-membrane microfluidic pump. The pumping action was stimulated by an electrochemical actuated PPy-PDMS membrane. The check valves were used to control the direction of flow. The maximum flow rate of 52 μL/min was obtained at ±1.5 V with input power of 55 mW.

#### 2.3.4. Evaporation Micropumps

**Figure 14:**
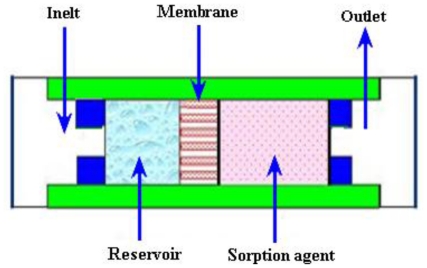
Evaporation micropump.

In evaporation micropumps, a controlled evaporation of liquid is used. Evaporation is a process in which liquid is converted from its liquid form to vapor form. The reverse of this process is known as condensation. The pumping principle of the evaporation type micropump is the same as the xylem transport system in plants. A schematic of an evaporation micropump is shown in Figure 14.

Effenhauser *et al*. [97] reported the evaporation based disposable micropump for continuous monitoring systems. The controlled evaporation of liquid was done through a membrane into gas space that contained a sorption agent. In the gas chamber, the vapor pressure was kept lower than saturation. During this process, the fluid evaporation from membrane was substituted by capillary forces that resulted in a flow from the reservoir. The average flow rate of 0.35 μL/min was achieved. Namasivayam *et al*. [98] reported the micropump based on the generally observed phenomenon of transpiration in plant leaves for continuous very low flow rates. As the vapor diffused out due to heating, a new transport of liquid was supplied into the channel from a reservoir for steady state operation. Guan *et al*. [99] reported a micropump based on capillary-evaporation effects for a microfluidic flow injection chemiluminescence system. The average flow rate of 3.02 μL/min was achieved with an ambient temperature of 20–21 °C and relative humidity of 30–32% for fluctuation within 2 h. Heuck *et al*. [100] reported the evaporation-based micropump integrated into a scanning force microscope probe for the flow of liquid through its hollow cantilever and tip areas. A flow rate of 11 pL/s was obtained at room temperature.

#### 2.3.5. Bubble Micropumps

**Figure 15:**
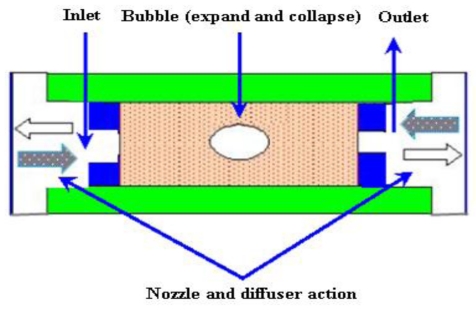
Bubble micropump.

The bubbles micropump is based on periodic expansion and collapse in the volume controlled by voltage input. The volume change in chamber is incorporated with the diffuser/nozzle mechanism that is used to determine the direction of fluidic flow. The bubbles are generated by heating process. A schematic of the bubble micropump is shown in Figure 15.

Tsai and Lin [101,102] reported a valveless thermal-bubble micropump. Later they developed a microfluidic mixer system with a gas bubble filter using the bubble micropump. The maximum flow rate of 5 μL/min was achieved at 250 Hz with applied periodic voltage, 10% duty cycle and power consumption of 1 W. Lew *et al*. [103] developed a collapsing bubble micropump. The bubbles with a radius of about 3–5 mm were investigated through the experimental set up that employed a low voltage electrical spark of 55 V created with a capacitor for bubble generation. It was reported that the proposed theory could also work with even smaller bubbles. Jung and Kwak [104] reported the fabrication and testing of bubble type micropumps using an embedded microheater. The micropump comprised of a pair of nozzle/diffuser, flow controller, microchannels and a pumping chamber. The maximum flow rates of 6 μL/min at duty ratio of 60% for circular chamber and 8 μL/min at duty ratio of 40% for the square chamber were achieved. Cheng and Liu [105] reported an electrolysis-bubble micropump based on the roughness-gradient design in the microchannel. The electrolysis actuation and the surface tension effect were used for the micropump. The maximum flow rate of 114 μL/min was obtained at applied voltage of 15 V with a frequency of 4.5 Hz. Chan *et al*. [106] developed a bubble type micropump with high frequency flow reversal using embedded electrodes in a closed microfluidic microchannel. The micropump consisted of a microfluidic chamber and microelectrodes on a glass substrate that was assembled by PDMS-sheet. The maximum flow rate of 37.8 μL/min was achieved at voltage of 5 V.

#### 2.3.6. Magnetohydrodynamic (MHD) Micropumps

MHD is a field in which the dynamics of electrically conducting fluids is studied. The Lorentz force is the driving source perpendicular to the electric and magnetic fields for MHD type of micropumps. The working fluid is selected to achieve conductivity of 1 s/m or higher, in addition to externally providing electric and magnetic fields. The Lorentz force can be expressed by the equation (11).

(11) F=QE+Q(V×B)

Where, *F* is force, *E* is electric field, *V* is instantaneous velocity of particles, *B* is magnetic field and *Q* is electric charge of the particle.

**Figure 16:**
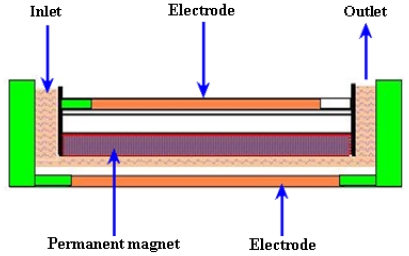
MHD micropump.

A schematic of the MHD micropump is shown in Figure 16.

Jang and Lee [107] reported the MHD micropump. The pressure head difference of 18 mm at 38 mA and a flow rate of 63 μL/min at 1.8 mA were achieved with an inside diameter of 2 mm for inlet/outlet tube and a magnetic flux density of 0.44 T. Zhong *et al*. [108] reported the fabrication of MHD micropump using ceramic tapes. Experiments were performed using mercury slugs, saline solutions and DI water. Eijkel *et al*. [109] developed a circular ac MHD micropump for chromatographic applications. The device comprised of a glass-gold-laminate-glass sandwich structure with the channel defined in the electroformed gold layer. Reversible flow rate of 40 μm/s was achieved. Patel and Kassegne [110] reported a MHD micropump with EO-thermal effects using 3D-MHD equations. The use of a developed numerical framework, flow channel geometries, Joule heating, effects of non-uniform magnetic/electric fields and EO in MHD micropumps were investigated. Duwairi and Abdullah [111] developed a model to envisage the fluid flow in the MHD micropump. By applying the finite difference method and the SIMPLE algorithm, the transient, incompressible, laminar and flow equations were numerically solved. Kang and Choi [112] reported the design and fabrication of MHD micropump with a mixing function in which the fluids were mixed and pumped at the same time by coupling between Lorentz force and the moving force of an electric charge in the electric field.

#### 2.3.7. Flexural Planer Wave (FPW) Micropumps

**Figure 17:**
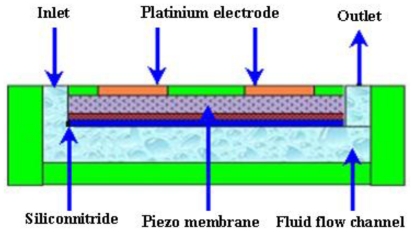
FPW micropump.

The FPW micropumps are driven ultrasonically. The fluidic motion induced by traveling FPW can be used for the transport of liquids. The liquid motion is in the direction of wave propagation and the speed is proportional to the square of acoustic amplitude. Low operating voltage is required for acoustic streaming. A schematic of the FPW micropump is shown in Figure 17.

Moroney *et al*. [113] reported the process of water pumping induced by 4.7 MHz ultrasonic Lamb waves. The waves were moving in a composite membrane of silicon nitride and piezoelectric zinc oxide with a thickness of 4 μm. The observed speed was 100 μm/s at the applied rf voltage of 8 V with 6.5 nm wave amplitude. Nguyen and White [114] reported the design and numerical model of an ultrasonic FPW micropump and microfluidic system. The effects of channel height, wave amplitude, and backpressure on the velocity and flow rate were studied. The influence of thermal transport of the acoustic streaming was also investigated. Results showed that the micropumps with channel heights of a few micrometers exhibited high-quality performance because the flow rate and hydraulic impedance against backpressure were high. Nguyen *et al*. [115] reported a FPW micropump integrated with flow sensor for *in situ* measurement. The FPW micropump and the flow sensor made a complex microfluidic system capable of controlling the fluid flow in the device. Meng *et al*. [116] reported the ultrasonic FPW micropump. The waves travelled along a thin membrane to stimulate an acoustic field in the fluid that was in contact with the membrane. The micropump with a combination of radial transducers and unidirectional fluid flow resulted in a flow speed of 1.15 mm/s. Jang *et al*. [117] investigated the actuating frequency control of acoustic-streaming flow patterns in a diaphragm driven microfluidic chamber. Microfluidic circulatory flow was achieved using the resonant vibration of diaphragms. Experiments were performed to study in-plane velocity profiles near the interface of circulations where the acoustic intensity was measured to be large. The proposed flow process was reported to be useful for pumping, active mixing and particle focusing applications. Singh and Bhethanabotla [118] studied the enhancement in the efficiency of acoustic-streaming. Microfluidic and biosensing applications of surface-acoustic wave devices depend on the acoustic-streaming process resulting from high intensity sound waves that interact with the fluid medium.

#### 2.3.8. Electrohydrodynamic (EHD) Micropumps

In an EHD micropump, the force is generated by the interaction of electric field and mobile charges in the fluid. These pumps have emitter and collector electrodes that are regularly spaced along a microchannel and require no moving parts such as impellers, bellows or valves. The electrical charges generated from the electrodes mobilize according to the direction of the electric field that is built up by the electrodes and tract in the surrounding liquid molecules to move together by the ion dragging force. The force acting on the fluid is given by the equation (12).

(12) $$F=\frac{Id}{k}$$

**Figure 18:**
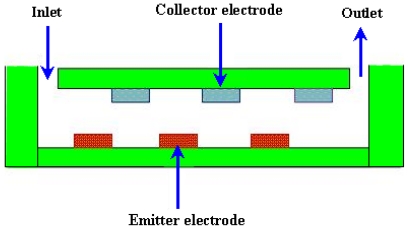
EHD micropump.

Where, *F* is force on fluid, I is current, d is distance between electrodes, k is ion mobility coefficient of the dielectric fluid. A schematic of an EHD micropump is shown in Figure 18.

Ritcher and Sandmaier [119] fabricated the first dc charged injection EHD micropump comprised of two electrically isolated grids. The flow rate of 15,000 μL/min and the pressure head of 1.72 kPa were achieved at applied voltage of 800 V. Fuhr *et al*. [120] developed the first EHD micropump based on travelling wave-induced electroconvection. The flow rates of 0.05–5 μL/min were achieved. Darabi *et al*. [121,122] reported the EHD polarization micropump for electronic cooling and EHD ion drag pump. The model devices exhibited a maximum cooling capacity of 65 W/cm^2^ with pumping head of 250 Pa. Yang *et al*. [123] reported an ejection type EHD micropump using indium-tin-oxide (ITO) planar electrodes to deal with the aging problem. The planar electrodes could drive the ethyl alcohol with a flow rate of 356 μL/min at applied dc voltage of 61 V. Lin and Jang [124] reported the numerical microcooling analysis for EHD micropump. The micropump offered the pumping power using the dipole moment force generated from polarizing fluid molecules. The pressure head of 13 kPa and wall heat flux of 10 W/cm^2^ were observed at applied voltage of 500 V with pitch of 500 μm for parallel electrodes. Darabi and Rhodes [125] reported the computational fluid model of ion drag EHD micropump. The micropump consisted of an array of interdigitated electrodes with the top and bottom parts of the channel. Singhal and Garimella [126] reported induction based EHD micropump for high heat flux cooling process. The numerical model was developed by solving the three dimensional transient fluid flow and charge transport problem due to simultaneous actuation of EHD and the vibrating diaphragm. Recently reported non-mechanical micropumps are listed in Table 2.

## 3. Microneedles

Microneedles are very useful delivery devices. These devices provide an interface between the drug reservoir and the patient’s body for releasing or extracting the fluid. The length of microneedles should be long enough that it penetrates the epidermis and short enough not to reach the dermis, in order to avoid pain. The concept of microneedles was proposed in the 1970s but it was not realized experimentally until the 1990s when the industry of microelectronics provided the microfabrication tools essential to make such small structures. The first microneedle arrays reported in the literature were developed by etching the Si wafer for intracellular delivery [132]. These needles were inserted into cells and nematodes to increase molecular uptake and gene transfection. After that a number of attempts have been made by various researchers to develop the fabrication processes and different designs of microneedles. MEMS technology is the most promising to fabricate the optimal design of microneedles for particular applications. The typical diameter and length of MEMS-based microneedles are in the range of micrometers. These microneedles are different from standard hypodermic needles used in biomedicine. Generally, the length of the MEMS-based microneedles is less than 1 mm. Thus microneedles are significantly smaller in length than ordinary needles [4,133]. Microneedles or microneedle arrays can be used as a stand-alone microfluidic device as well as part of biological detection, fluid extraction or delivery system. Microneedles can be integrated with micropumps, biosensors, microelectronic devices and microfluidic chips.

### 3.1. Categories of Microneedles

Different designs of microneedles have been reported in literature for various applications. Microneedles can be classified in various ways such as according to structure, overall shape, tip shape, length, array density, material used for fabrication and applications [3,4]. Details of microneedle categories are shown in Table 3.

#### 3.1.1. Structure of Microneedles

Structure is the most important consideration for microneedles design and fabrication. Based on the fabrication process, the microneedles are classified in two types.

- In-plane microneedles
- Out-of-plane microneedles

**Figure 19:**
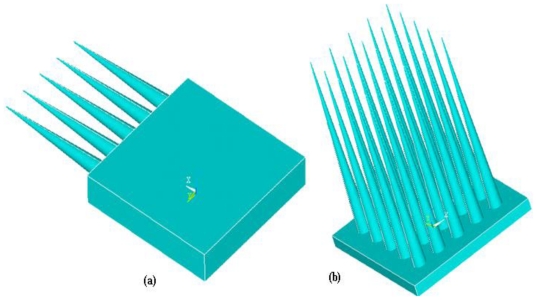
(**a**) In-plane microneedles; (**b**) Out-of-plane microneedles.

In in-plane microneedles, the microneedle shafts or lumens are parallel to the substrate surface. The major advantage of in-plane microneedles is that the length of the microneedles can be easily and accurately controlled during fabrication process. The limitation of in-plane microneedles is that it is very difficult to fabricate microneedle arrays with 2D geometry. In out-of-plane microneedles, the lengths of the microneedles protrude out of the substrate surface and it is easier to fabricate out-of-plane microneedles in 1D or 2D arrays. However, fabrication of out-of-plane microneedles with length and high aspect ratio structure is challenging [4,134]. A schematic illustration of in-plane and out-of-plane microneedles is shown in Figure 19.

In-plane microneedles were developed in the 1980s [134] and not intended for drug delivery or fluid transport. An implantable ten-channel microelectrode recording array with an on-chip signal processing probe was fabricated for long term recording of neural bio-potentials. The length of probe and thickness were 4.7 mm and 15 μm respectively. A 1D array of micro neural probes [135] and more sophisticated 2D array have been developed [136]. After that various attempts have been made to develop in-plane microneedles for different applications. The major drawback associated with in-plane microneedles is the limited density. To overcome this limitation, out-of-plane microneedles have been developed. One of the earliest out-of-plane microneedle array consisted of 100 microneedles with a length of 1.5 mm was reported in 1991 [132].

**Figure 20:**
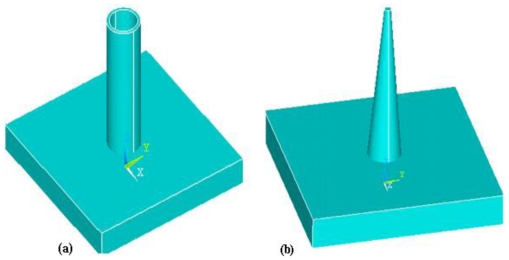
(**a**) Hollow microneedle; (**b**) Solid microneedle.

Microneedles can also be categorized as solid or hollow according to the structure. Hollow needles were invented in 1844 [137] and gained increasing importance in the biomedical field. There are no other effective ways to transport the fluid into the human body [138]. Hollow needles have become more important after the invention of microneedles. Hollow microneedles have an internal bore or lumen which allows flow of fluid/drug through the microneedles. A combination of surface and bulk micromachining techniques was used to fabricate hollow in-plane microneedles with 1-6 mm length and fully enclosed channels of silicon nitride [139]. The channels were 9 μm in height. The solid microneedles have solid lumens and exhibit more strength than hollow microneedles. Solid microneedles can be further categorized into coated and dissolving microneedles. In coated microneedles, the drug particles are coated on lumen surface and injected into patient body. The microneedles are withdrawn from the body after dissolution of the coated drug. In dissolving microneedles, the base is non-dissolving and withdrawn from the skin after dissolution of the microneedles. Various types of solid coated and dissolving microneedles have been reported [19]. Coated Ti microneedles arrays with a length of 190 μm have been reported for the delivery of parathyroid hormone (I—34) in human body for the treatment of osteoporosis by Zosano Pharma, Inc. (formerly Macroflux®, ALZA Corp.) [140]. The successful delivery of drug depends on the methods used for coating of microneedles [141,142]. References [143,144] fabricated the first out-of-plane sharp solid microneedles for drug and gene delivery. A schematic of hollow and solid microneedles is shown in Figure 20.

Hollow silicon dioxide (SiO_2_) microneedles have been fabricated using deep reactive ion etching technique [145]. Reference [146] fabricated SiO_2_ microneedles which mimic a jagged mosquito’s needle. In-plane hollow metallic hypodermic microneedles and microneedle array were reported using electroplated palladium (Pd) alloys and Ni [147–149]. Using a combination of isotropic and an isotropic etching process, sharp tip hollow out-of-plane single crystal Si microneedles were fabricated [150]. One of the earliest solid microneedles design was in the form of pyramidal Si microprobes [151]. Sharp Si solid microstructures with a height of 150 μm were fabricated with anisotropic dry etching technique using SF_6_ and O_2_. Such type of solid microneedles was used to increase permeability of human skin up to fourth order of magnitude *in vitro*. Solid microneedles for TDD were reported for the first time in 1998 [144].

#### 3.1.2. Shape of Microneedles

**Figure 21:**
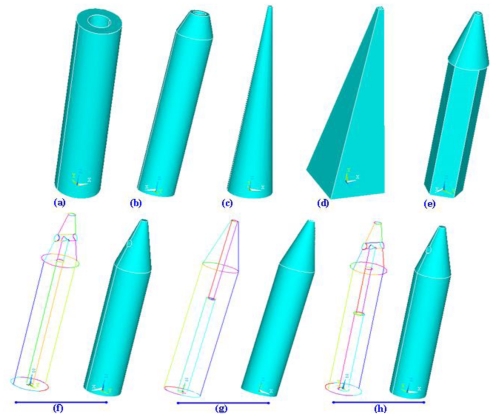
Shapes of microneedles (**a**) Cylindrical; (**b**) Tapered tip; (**c**) Canonical; (**d**) Square base; (**e**) Pentagonal-base canonical tip; (**f**) Side-open single lumen; (**g**) Double lumen; (**h**) Side-open double lumen.

The shape of the microneedle is very critical and important during design and fabrication. Microneedles can be classified on the basis of overall shape and tip shape. Different designs of microneedles have been proposed and fabricated such as cylindrical, canonical, pyramid, candle, spike, spear, square, pentagonal, hexagonal, octagonal and rocket shape [3,4]. Microneedles have also been reported with various tip shapes like volcano, snake fang, cylindrical, canonical, micro-hypodermis and tapered. Schematic illustrations of various designs of microneedles with respect to shape and tips are shown in Figure 21.

Rocket shape microneedles have been fabricated using two photon polymerization method [152]. Octagonal solid out-of-plane Si microneedle array has been fabricated for drug delivery [153]. Solid Si-tip microneedles have been fabricated using wet etching technology [154]. Pyramidal out-of-plane Si microneedle array has been fabricated by wet etching for transcutaneous drug delivery [155]. Side opened sharp tip out-of-plane solid microneedle has been fabricated by hot embossing to improve skin permeability for hydrophilic molecules [156]. Cylindrical hollow out-of-plane microneedles with tapered tip using combination of ICP etching have been fabricated for TDD [4].

#### 3.1.3. Materials Used for Microneedles

Microneedles can be classified on the basis of materials. Material selection is very important to design and fabricate microneedles for any particular application. Many researchers used Si for microneedles fabrication [4,157–162], which is a brittle material [163] and can be harmful to health. Different researchers have understood this critical issue and used polymeric material instead. Most polymers have a strong history of biocompatibility. They exhibit excellent mechanical and chemical properties [164] that are suitable for microneedle fabrication. Fabrication of microneedles has been reported using various polymers such as (Polyglycolic acid) PGA, (Poly-L-Lactide acid) PLLA, PC, PDMS, PMMA, *etc*. Fabrication of polymeric microneedles has been reported by various researchers [165,166]. Some other materials have also been reported such as glass, metal, alloy, *etc*. [4]. Glass hollow elliptical tip microneedles have been fabricated using micropipette pulling technique for intrascleral delivery [167]. In-plane Ti microneedles have been fabricated using bulk micromachining for drug delivery [168]. Tungsten microneedles have been reported for nerve penetration [169].

#### 3.1.4. Microneedles Applications

On the basis of applications, microneedles can be categorized into various types because different types of microneedles are suitable for specific applications. The suitable length of microneedles for drug delivery is 100 μm to 300 μm, but for blood extraction the appropriate length of microneedles is 1100 μm to 1600 μm [170]. Solid microneedles are suitable for cell surgery. Microneedles have been reported for drug delivery, blood extraction, fluid sampling, cancer therapy, microdialysis, ink-jet printing and sensing electrodes. Hollow Ti microneedles have been fabricated for blood extraction using sputtering and deposition methods [171]. SiO_2_ hollow square microneedles have been reported for flow delivery systems using electrochemical etching technique. Hollow out-of-plane SiO_2_ microneedles have been fabricated using lithography for cell surgery [162]. Stainless-steel hollow and solid microneedles have been reported using surface micromachining and etching techniques for dermal diphtheria and influenza vaccination [172]. Hollow out-of-plane Si microneedles have been fabricated for TDD [4]. The extensive detail of materials used for microneedle’s designs, structure, array size, fabrication techniques, analysis and application has been presented in Table 4, Table 5 and Table 6.

### 3.2. Forces Experienced by Microneedles during Penetration

Fluid is transported through hollow microneedles while solid microneedles are coated with pharmaceutical materials to transfer the drugs into patient body. Microneedles are under the influence of various forces during penetration such as bending, buckling, lateral, axial and resistive. To bear all these forces, the design of microneedles is very important. Microneedles can break during penetration into the skin because of these forces. An axial force is more dominant on the tip of microneedle during insertion. This axial force is compressive and leads to buckling of the microneedle. The microneedles also experience resistive force exerted by skin. Hence, in order to pierce the microneedle into skin, the applied axial force must be greater than skin resistance. Due to uneven skin surface or human error during needle penetration, bending may occur. So, it is very important to study the relation between microneedle geometry and mechanical properties of the material for accurate microneedle design and prediction of microneedles failure. The buckling force acting on the hollow microneedle during skin insertion is given by [4,133,196,197].

(13) $$B_{\text{Buckling}}=\frac{\pi^{2}EI}{L^{2}}$$

Where, E is Young’s modulus of material, I(m^4^) is moment of inertia of cylindrical section and L(m) is length of the microneedle.

Moment of inertia (I) for hollow cylindrical section of microneedle is calculated by equation (14).

(14) $$I=\frac{\pi }{64}(D^{4}+d^{4})$$

Where, D is outer diameter and d is inner diameter of hollow cylindrical section.

The bending force, which the microneedle can withstand without breaking is given by:

(15) $$F_{\text{Bending}}=\frac{\sigma_{yI}}{cL}$$

Where, $\text{C}=\frac{\text{D}}{2}$ is the distance from vertical axis to the outer edge of the section [3,4,198].

The axial force (compressive force), which a microneedle can withstand without breaking is given by:

(16) $$F_{\text{Compressive}}=\sigma_{y}A$$

Where, σ_y_ is the yield strength of the material and A is cross-sectional area of the microneedle tip.

Microneedle experiences 3.18 MPa resistive forces exerted by the skin against penetration of microneedle. To penetrate the microneedles into skin, the external applied force or pressure should be greater than the resistive skin force. The resistive force offered by the skin before puncturing is given by the following equation:

(17) $$F_{\text{resistance}}=P_{\text{pierce}}A$$

Where, P_pierce_ is the required pressure to pierce the microneedle into skin.

As the microneedle penetrates the skin, the resistive force falls drastically [199]. After the skin is pierced by the microneedle, the only force that acts on the microneedles is the frictional force due to contact of tissue with the microneedle.

### 3.3. Fabrication of Microneedles

Various fabrication techniques have been developed and used for microneedles fabrication such as hot embossing [156], photolithography [162], micropipette pulling technique [167], surface micromachining [172], bi-mask technique [175], laser micromachining [179], micro-molding [181], deep x-ray lithography [200], DRIE [176], lithography, electroplating, molding (LIGA) [201], UV excimer laser [202], coherent porous Si etching (CPS) [203], injection molding [204] and ICP etching [3,4]. In these processes, Si and polymer can be used as substrate materials for microfabrication. Each fabrication technique has its own advantages and limitations. A detailed discussion on microneedles fabrication techniques will be presented in a subsequent paper.

Lithography and DRIE techniques are mostly used for fabrication of silicon microneedles. Deposition and etching are the most important phenomenon during the development of microneedles. Deep holes or free standing structures can be fabricated in silicon wafer with the help of anisotropic etching process. These high aspect ratio structures are of considerable interest in developing micro devices for various applications. The general steps of silicon microneedles fabrication are wafer cleaning, photoresist coating, soft back, masking, exposure, development, hard back, and lift off. These steps can be repeated according to requirements with desired parameters. For polymeric microneedles molding, hot embossing, and laser drilling are promising fabrication techniques. The general steps for polymeric microneedles fabrication are sheet preparation, mold preparation, heating and pressing mold, de-molding and laser drilling for different lumens/reservoir.

### 3.4. Microneedles Testing

The concept of microneedles was introduced three decade ago but the first microneedle array for TDD was fabricated in 1998. After that, various researchers have been involved in developing the most suitable fabrication method and optimal design of microneedles for biomedical applications. After 2005, the interests of researchers changed and they shifted their attention towards the testing of microneedles along with design and fabrication. Most of the research groups have been involved in the structural analysis and skin penetration tests of microneedles in 2010. The testing of microneedles has been reported on potato skin, chicken skin [175], mouse skin [156,172,180,187,193], cadaver skin [167,182], pig skin [173,177], chicken leg, beef liver [204], and human skin [156,174,178,183]. Microneedle patches coated with solid state influenza vaccine have been reported to improve the effectiveness of the vaccine when tested on mouse skin [190]. Dry coated microinjection arrays have been developed to deliver HSV-2-gD_2_ DNA vaccine to sensitive regions of mouse skin [205]. The pretreatment of skin by microneedles was combined with the use of highly water soluble pegylated naltrexone for its transdermal delivery at different concentrations [206]. A new design of probe integrated with hollow microneedles for atomic force microscope (AFM) has been developed to realize cellular function analysis in a single living cell [176]. The geometry of microneedles affects its strength. The shear strength of hollow silicon microneedles can be increased by variation in microneedles geometry [207]. Using novel microneedle technology, hydrophobic dye called Nile red has been delivered into porcine skin [169]. The effect of microneedle geometry has been studied on the transport of a fluorescent dye into human skin [208]. To envisage the effect of microneedle geometry and force of application, optical coherence tomography has been used by penetrating microneedles arrays into neonatal porcine skin [209]. Short densely packed microinjection array has been developed to see the effect of strain rate on the precision of penetration into human skin [210]. Reference [211] has investigated that influenza virus like particles coated on microneedles can cause stimulatory effect on langerhans cells in human skin. The super short microneedles have been fabricated using Si wet etching technology and tested for TDD into human skin [212]. The separable arrowhead microneedles have been introduced and tested for painless delivery of drugs and vaccines into human cadaver skin [213]. A minimally invasive system has been developed using microneedle electrode array to deliver macromolecular drugs to the deep skin tissues and tested on hairless rat skin [214]. Solid silicon microneedles arrays have been used with different lengths and geometry to penetrate epidermal membrane of human cadaver skin [215]. The microneedles coated with influenza virus like particles have been used to test the immunogenicity and protective efficacy after vaccination of mice skin [216]. Microneedles rollers have been developed and tested on human and porcine skin to increase skin permeability and to treat skin for cosmetic purposes [167]. Microneedles have been used to deliver PLGA (poly-lactic-co-glycolic-acid) nanoparticles in the human skin [174]. Solid polymeric microneedles have been developed to investigate the transepidermal water loss measurements of dermatomed human skin [183]. The efficacy of transdermal delivery of insulin has been investigated by using microneedles rollers on diabetic rats [217]. The administration of virus like particles influenza vaccine has been studied using microneedles patch on lungs and bone marrow cells of mouse [218]. Hollow microneedles array with sonophoretic emitter has been used on pig skin to improve the efficiency of drug delivery [173].

## 4. Discussion

**Figure 22:**
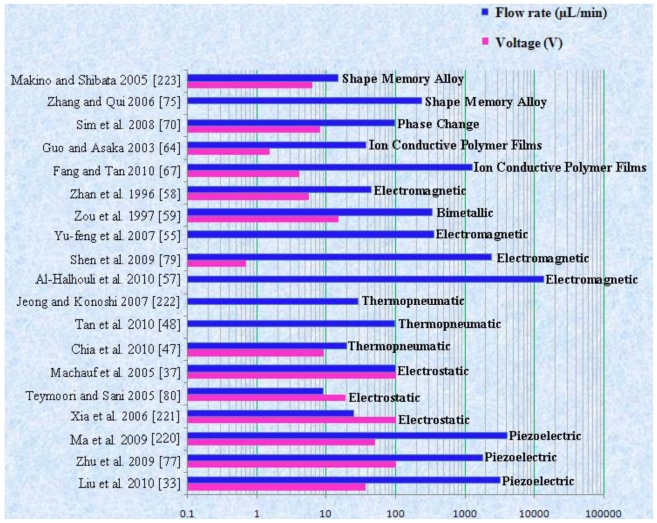
Comparison of voltage *versus* flow rate for mechanical micropumps.

MEMS and NEMS based microfluidic devices have many important characteristics that make them attractive for biomedical applications. Microfluidic devices have the ability to control their physical and chemical characteristics from a very small scale up to the nanometer range. These devices have made it possible to meet critical medical needs such as nearly constant drug level at the site of action, prevention of peak-valley fluctuations, site specific drug delivery, reduced side effects and increased therapeutic effectiveness [4]. However, there are certain medical conditions for which constant drug release pattern is not suitable. These conditions demand delayed release of drug. Such a release pattern is known as pulsatile release. Recent research has shown that some diseases have a predictable cyclic rhythm and the timing of drug release can significantly improve the outcome of a desired effect [219]. This condition requires release of drug as a pulse after a time delay. Some of the diseases where pulsatile drug delivery devices are promising include duodenal ulcer, cardiovascular diseases, arthritis, asthma, diabetes, neurological disorder, cancer, hypertension and hypercholesterolemia [4]. That is why study of pulsatile flow is extremely important at small scales in microfluidic devices. Using MEMS and NEMS technology, complex drug release patterns (such as simultaneous constant and pulsatile release) can be achieved using integrated microfluidic systems. Microfluidic devices have ability to control both release time and release rate. Micropumps and microneedles are essential components for such biomedical systems. Micropumps are used for fluid transport and microneedles provide interface between drug reservoir and patient body [4,13,6]. Material selection is a critical issue in biomedical devices. Si has been widely used as material for such microfluidic devices, but polymeric materials like PGA, PDMS, PMMA, PLLA, PLA, PC, *etc*. are replacing Si due to biocompatibility, low cost, ease of fabrication and excellent structural properties. Various factors are important during the selection of micropumps for particular biomedicine applications. Operating voltage, pressure and flow rate of micropumps are critical issues to analyze the performance and suitability of micropumps for certain medical applications. A schematic illustration of operating voltages and flow rates of mechanical micropumps is shown in Figure 22.

**Figure 23:**
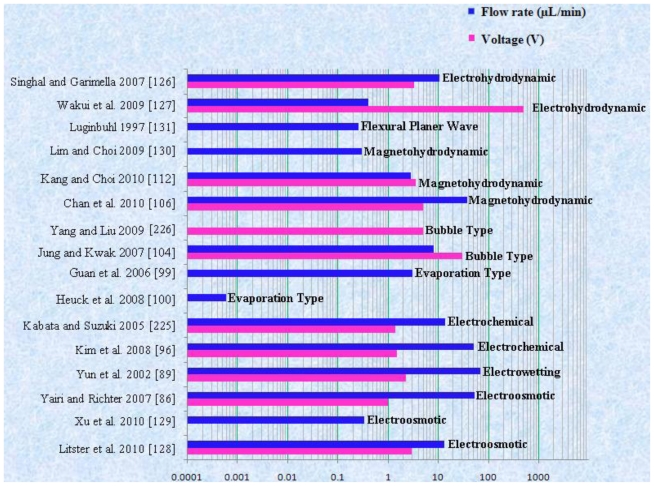
Comparison of voltage *versus* flow rate for non-mechanical micropumps.

Piezoelectric and electromagnetic mechanical micropumps have been reported extensively for microfluidic systems among the mechanical micropumps. The major limitation related to these types of micropumps is very high operating voltage [224]. Electrostatic micropump is easy to fabricate on integrated microfluidic systems but it also requires high operating voltage. The ICPF micropump has an adequate flow rate at a relatively low operating voltage but with complex geometry. The schematic illustration of operating voltages and flow rates of non-mechanical micropumps is shown in Figure 23.

**Figure 24:**
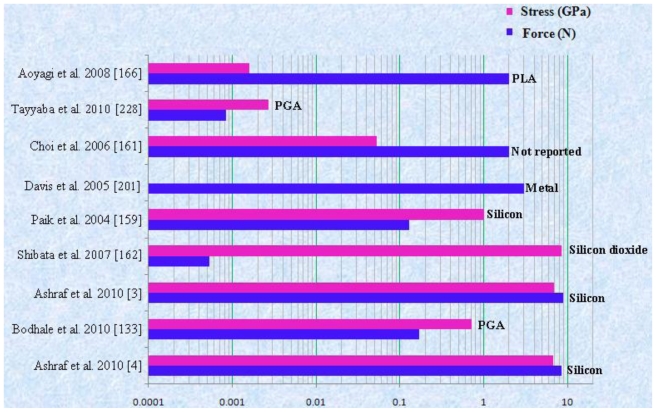
Comparison of force *versus* stress on microneedles.

In non-mechanical micropumps, MHD micropump has gained more attention in recent years and has been presented by many researchers for microfluidic systems. However, the electrochemical type of micropump is more suitable for low voltage and high flow rate applications. Most micropumps and microneedles have been reported in literature as an individual device for medical applications. Only a few researchers have presented integrated devices [227]. Integration of micropumps and hollow microneedles is a great challenge, but research on solid microneedles coated with nano-particles and drugs has recently commenced in the biomedical field. However, hollow microneedles are more attractive for fluid/drug transport. Microneedles can be integrated with micropumps or used as stand-alone biomedical device. Various types of microneedles have been presented by different researchers. In hollow microneedles, the side opened double lumen reservoir based microneedles are more suitable for fluid transport. The pressure difference in the lumen regions is useful to avoid the clogging effect. In solid microneedles, the sharper tip microneedles are more practical for drug transport. Effectiveness of drug transport has also been presented in recent years by various researchers using microneedles on mouse, pig, chicken and human. Various researchers have reported the structure and fracture analyses of microneedles by applying force and pressure to predict the bending and failure of microneedles. The schematic illustration of comparison of force and stress for microneedles has been shown in Figure 24.

## 5. Challenges and Future Aspects

In biomedical field, there are many challenges relating to the microfluidic devices (micropumps, microneedles) such as design level issue, fabrication level issue, packaging level issues, use in practical application, *etc*. At design and fabrication level, the most important issues and specifications that must be fulfilled by the micropumps for particular applications are appropriate design for maintaining a specific flow rate, control of back pressure, dosing accuracy, drugs resistive material selection for fabrication, ease of fabrication, energy utilization, power supply at such small level, bubble tolerance, durability and reliability. The most important issues relating to microneedles at design and fabrication level are avoidance of clogging effect, suitable length, robustness, strength, sharp tip to avoid pain, drugs resistance, less fabrication cost, reliability, biocompatibility, *etc*. Suitable batch fabrication techniques need to be adopted to reduce cost of devices. Packaging of these devices is very important consideration [229]. Packaging should be robust and strong enough to prevent infection or damage of microfluidic devices. Simultaneously, the unintentional discharge of drug/fluid during storage from the reservoir should be prevented. A protecting wrap may be possibly required to secure such small size devices like micropumps and sharp tip microneedles. Mostly the micropumps and microneedles reported in literature have been proposed as stand-alone devices. Integration of micropump and microneedles is a great challenge that limits the use of these devices commercially for biomedical applications. The final cost of these delivery devices should be affordable for the end users/patients. The trend is now shifting towards the use of polymeric materials like PGA, PDMS, PMMA, *etc*. for the fabrication of micropumps and microneeldes to overcome most of the above described issues as these materials are cheap, biocompatible, exhibit excellent mechanical/chemical properties, *etc*. [4,13,19,202]. Zosano Pharma [140] has developed the user friendly and simple TDD patch system that can deliver vaccines, proteins, peptides and small molecules. 3 M has developed the microneedles based transdermal system demonstrating good results on research level studies for peptides, vaccines and protein [230]. Birchall *et al*. [231] conducted a survey to learn more about the opinion of end users with regard to the convenience, efficacy and worth of microneedle technology. Research on MEMS-based delivery devices shows that these devices are suitable for commercial applications. However, the development of these devices is limited to research level due to some factors such as investment, expertise for device development, marketing, awareness of public, motivation, lack of collaboration between companies and research institutes, medical staff training/recommendations, *etc*. Surveys, seminars, workshops, *etc*., need to be organized to promote the benefits and convenience of using these delivery devices to end users. The availability of reliable and manufacturable microfluidic devices will have a strong impact on the biomedicine field to meet critical health care needs.

## 6. Conclusions

Fluid transport using microfluidic devices such as micropumps and microneedles is a relatively new and attractive method that has many advantages. Microfluidic devices have received much more attention in recent years due to their potential applications in the biomedicine field. Various types of micropumps and microneedles structures using different materials like glass, silicon, metals, polymers, *etc.*, have been reported for biomedical applications, but Si has been mostly used as substrate material for fabrication of microfluidic devices among other materials. Si is brittle and always some risk involves for health care. Biocompatibility is very important for health and because of this reason the trend is transferring towards polymeric materials. Most polymers, e.g., PGA, PDMS, PLA and PMMA, are very suitable for biomedical devices due to their good biocompatibility, low cost, ease of fabrication and excellent chemical and mechanical properties. Solid microneedles are easy to fabricate and have more strength than hollow microneedles. However, the disadvantage of solid microneeldes is the risk of fracture/breaking within the skin after being inserted. Drug particles can be coated in restricted amounts. Hollow microneeldes are considered more suitable for TDD systems due to the precise delivery of the desired amount of drug at a specific site with rapid action. ICPF and electrostatic micropumps are considered suitable for drug/fluid delivery systems due to low operating voltage and direct integration with electronic circuit respectively. The disadvantages of hollow microneeldes and micropumps are complicated/costly fabrication, clogging effect, back pressure, requirement of flow rate regulation, *etc*. Based on the presented literature review, the authors conclude that MEMS based microfluidic devices for biomedical applications remain at the research level. Only a few devices have been converted into commercial products due to some important issues like complicated structure of microfluidic devices, difficulties in integration with other devices, investment, expertise for device fabrication, marketing, public awareness, lack of collaboration between companies and research institutes, medical staff training/recommendation and finally packaging. To present microfluidic devices for practical applications in medical field, motivated researchers still need to continue their work on the development of microfluidic devices.

## Figures and Tables

**Table 1:** Recently reported mechanical micropumps.

Reference	Actuation Method	Materials used for fabrication	Size	Pumping Chamber	Pumping Medium	Valve	Voltage (V)	Frequency	Back Pressure/Applied Pressure	Flow Rate (μL/min)	Applications
**Liu*et al*. 2010 [33]**	Piezoelectric	Polycarbonate (PC), PMMA, PDMS, PZT, (Titanium) Ti	15 × 8 mm	4	Insulin	2	36	200 Hz	22 kPa	6.23 × 10^−5^ mL/min	Insulin therapy system
**Zhu*et al*. 2009 [77]**	Piezoelectric	Polyetheretherketone/PDMS/Metal/Ceramics	Not reported	1	Air/Water	2	100	225 Hz for air, 17 Hz for water	Not reported	39 mL/min for air, 1.8 mL/min for water	Drug delivery applications
**Kang and Auner 2011 [78]**	Piezoelectric	Si/Epoxy H31/PZT-5A	14.5 × 9 × 1.1 mm	1	Not reported	2	240	20–100 Hz	0–10 psi	0.52 mL/min	Microfluidic applications
**Halhouli*et al*. 2010 [57]**	Electromagnetic	PC, Plexiglass	16 × 18 mm	1	Water	Not report ed	Not reported	Not reported	785 Pa	13.7 mL/min	Biomedical applications
**Shen*et al***. **2009 [79]**	Electromagnetic	PDMS, Glass	24 × 40 × 0.4 mm	3	Water	2	0.7	12 Hz	70 mbar	2.4 mL/min	Portable LOC applications
**Lee*et al*. 2009 [39]**	Electrostatic	Si	Not reported	2	Gas	19	Not reported	2.2–2.8 KHz	7.3–3.3 kPa	0.29–0.07 SCCM	Not reported
**Teymoori and Sani 2005 [80]**	Electrostatic	Si, Glass	7 × 4 × 1 mm	3	Not reported	3	18.5	50 Hz	Not reported	9.1 μL/min	Drug delivery applications
**Chia*et al***. **2010 [47]**	Thermopneumatic	PDMS, Glass	16 × 18 × 5 mm	3	Not reported	Not reported	9	1.2 Hz	490 Pa	20.01 μL/min	Biomedical applications
**Tan*et al***. **2010 [48]**	Thermopneumatic	PDMS, PMMA	Not reported	3	Compressed air	3	Not reported	10 Hz	138 kPa	96 μL/min	Microfluidic devics
**Zou*et al*. 1997 [59]**	Bimetallic	Al, Si, Glass	13 × 7 × 2 mm	2	Gas/Water	2	15	Not reported	0.5 kPa	5.6 μL/s	Not reported
**Fang and Tan 2010 [67]**	ICPF	PDMS, Polypyrrole, Stainless steel, Polyvinylidene fluoride	25 × 25 × 10 mm	1	Water	4	4	0.5 Hz	1.3 kPa	1260 μL/min	Biomedical devices
**Sim*et al*. 2008 [70]**	Phase Change	Al, Silicon, Silicone rubber, Glass	Not reported	1	Water	2	8	2 Hz	0 mm H_2_O	97 μL/min	Not reported
**Zhang and Qiu 2006 [75]**	SMA	Ti, Nickel (Ni), Copper (Cu)	8 × 8 × 1.8 mm	1	DI water	2	Not reported	80 Hz	Not reported	235 μL/min	Not reported

**Table 2:** Recently reported non-mechanical micropumps.

Reference	Actuation Method	Material used for Fabrication	Size	Pumping Chamber	Pumping Medium	Valve	Voltage (V)	Frequency	Back Pressure/Applied Pressure	Flow Rate (μL/min)	Applications
**Chan*et al*. 2010[106]**	Bubble type	PDMS, Glass, Si	Not reported	2	DI water, Phosphate- buffered solution	Not reported	5	300 Hz	Not reported	37.8 μL/min	Miniature electronic devices
**Jung and Kwak 2007 [104]**	Bubble type	Si, Pyrex glass	Not reported	1	DI water	Not reported	30	0.5–2.0 Hz	Not reported	6–8 μL/min	Microfluidic applications
**Wakui*et al*. 2009 [127]**	EHD	Polymer, Carbon, Glass	Not reported	Not reported	Fluorinert	Not reported	500	Not reported	23 Pa	400 μL/min	Microfluidic devices
**Singhal and Garimella 2007 [126]**	EHD	Al	1500 × 200 × 50 μm	1	Water	Not reported	3.3	373 kHz	Not reported	10.5 μL/min	Microchannel cooling system
**Lister*et al*. 2010 [128]**	EO	Glass, Platinum	Not reported	Not reported	Borate buffer, DI water	Not reported	2.9	Not reported	1.6 kPa	13 μL/min	Drug delivery
**Xu*et al*. 2010 [129]**	EO	Glass, PDMS	Not reported	2	Water	1	Not reported	Not reported	Not reported	0.33 μL/min	Perfusion cell culture
**Kang and Choi 2010 [112]**	MHD	Au (gold), PDMS	Not reported	Not reported	PBS solution	Not reported	3.6	Not reported	Not reported	2.83 μL/min	LOC applications
**Lim and Choi 2009 [130]**	MHD	Si, Pyrex glass, Al	40 × 25 × 1 mm	Not reported	PBS solution	Not reported	Not reported	Not reported	100000N/m^2^	0.3 μL/min	Drug delivery
**Yun*et al*. 2002 [89]**	EW	Glass, Si, Platinum	Not reported	2	Mercury	2	2.3	25 Hz	800 Pa	70 μL/min	Biomedical Devices
**Kim*et al*. 2008 [96]**	Electrochemical	Ppy, PDMS, PMMA	5.6 × 16 × 26 mm	1	Water	2	±1.5	Not reported	11 mbar	52 μL/min	Microfluidic applications
**Heuck*et al*. 2008 [100]**	Evaporation	Si	Not reported	Not reported	DI water	Not reported	Not reported	Not reported	Not reported	11 pL/s	Biological sampling
**Guan*et al*. 2006 [99]**	Evaporation	Pdms, PMMA, Stainless steel	25 × 15 × 3 mm	Not reported	Water	Not reported	Not reported	Not reported	23.5 kPa	3.02 μL/min	Microfluidics system
**Luginbuhl 1997 [131]**	FPW	Si, Platinum, Ceramic	Not reported	Not reported	Water	Not reported	6	2–3 MHz	Not reported	0.255 μL/min	Fluid delivery system

**Table 3:** Categories of microneedles.

Structure	Overall Shape	Tip Shape	Material Used	Application

Solid	Cylindrical	Volcano	Single crystal silicon	Drug delivery
Hollow	Canonical	Snake fang	Polysilicon	Gene delivery
In-plane	Pyramid	Cylindrical	Silicon dioxide	Blood extraction
Out-of-plane	Candle	Canonical	Silicon nitride	Fluid sampling
	Spike	Microhypodermis	PGA	Vaccination
	Spear	Tapered	PDMS	Micro-dialysis
	Square		PMMA	Cancer therapy
	Pentagonal		Glass	Dentistry
	Hexagonal		GaAs	Skin treatment
	Octagonal		Titanium	Cell surgery
	Rocket		Ti- alloy	Allergies diagnosis
	Star		Tungsten	Animal identification
			Tungsten-alloy	Ink-jet printing
			Stainless steel	Sensing electrodes

**Table 4:** Recent review of silicon microneedles.

Reference	Material	Structure of microneedles	Shapes of microneedles	Dimensions	Array size/Needles	Analysis type	Testing	Fabrication techniques	Application
Waseem *et al*. 2010 [4]	Silicon	Hollow/Out-of plane	Cylindrical	L = 200 μm D_i_ = 60 μm D_o_ = 150 μm	5 × 5	Structural/CFD (Static/Transient)	Not reported	ICP etching	Transdermal drug delivery
Chen *et al*. 2010 [173]	Silicon	Hollow/Out-of plane	Cylindrical base	L = 100 μm D = 80 μm	30 × 30	Fluidic analysis	Pig Skin	Deep reactive ion etching (DRIE)	Transdermal drug delivery
Zhang *et al*. 2010 [174]	Silicon	Solid/Out-of plane	Star shape	L = 200 μm	10 × 10	PLGA nano Particles distribution	Human skin	RIE/Thin film deposition Photolithography	Transdermal drug delivery
Waseem *et al*. 2010 [3]	Silicon	Hollow/Out-of plane	Cylindrical base tapered tip	L = 200 μm D_i_ = 40 μm D_o_ = 425 μm	6 × 6	Structural/Fluidic (Static/Transient)	Not reported	ICP etching	Transdermal drug delivery
Zhang *et al*. 2009 [175]	Silicon	Hollow/Out-of plane	Cylindrical/Side opened at tip	L = 200 μm D_i_ = 40 μm D_t_ = 450 nm	10 × 11	Fluidic analysis	Potato skin/Chicken skin	Bi-mask technique	Drug delivery/fluid sampling
Ding *et al*. 2009 [172]	Silicon	Solid/Hollow	Tangentially cut tip	L_1_ = 300 μm to 900 μm L_2_ = 300 μm D_2_ = 200 μm L_3_ = 245 μm	4 × 4 9 × 9	Fluidic analysis/Statistical	Mouse skin	Surface micromachining/Etching	Dermal diphtheria/influenza vaccination
Haq *et al*. 2009 [155]	Silicon	Hollow/Out-of- plane	Pyramidal	L_1_ = 180 μm L_2_ = 280 μm D_b_ = 180 μm	6 × 6	Fluidic analysis	Human skin	Wet etching	Transcutaneous drug delivery
Yu *et al*. 2009 [176]	Silicon	Hollow/Out-of plane	Cylindrical	D_P_ = 200 μm D = 100 μm	Not reported	Structural analysis	One-lead ECG recording system	DRIE	ECG measurement
Coulman *et al*. 2009 [154]	Silicon	Solid	Pyramidal shape/Pointed/Frustum tip	L = 280 μm, D_b_ = 200 μm	16 needles	Diffusion of nano particles	Human epidermal membrane	Wet etching	Transdermal/Intradermal drug delivery
Chen *et al*. 2008 [177]	Silicon	Out-of plane	Macro porous tip	Not reported	Not reported	Fluidic analysis	Pig skin	DRIE	Transdermal drug delivery
Roxhed *et al*. 2008 [178]	Silicon	Out-of- plane/Hollow	Cross/Circler	L_1_ = 310 μm, L_2_ = 400 μm	25 needles	Fluidic analysis	Human skin	DRIE	Transdermal drug delivery
Bhandari *et al*. 2008 [179]	Silicon	Hollow/Out- of-plane	Square base canonical	Not reported	10 × 10	Not reported	Not reported	Laser micromachining/Dicing/Etching	Blood sampling
Donnelly *et al*. 2008 [180]	Silicon	Not reported	Sharp 3D Tip/Grooves- embedded shaft	L = 270 μm, D_b_ = 240 μm	Not reported	Fluidic analysis/Statistical	Mouse skin/Porcine skin of piglets	Wet etching	Photodynamic therapy
Lee *et al*. 2008 [181]	Silicon	Solid/Out- of plane	Conical/Pyramidal	L_1_ = 800 μm, D_b_ = 200 μm, D_t_ = 20 μm, L_2_ = 600 μm, W_b_ = 300 μm	3 × 3	Structural analysis	Not reported	Micromolding	Drug delivery

**Notations:** L = Length of needle, W_b_ = Base width, D_o_ = Outer diameter, D_i_ = Inner diameter, D_b_ = Base diameter, D_t_ = Tip diameter, D_P_ = Depth.

**Table 5:** Recent review of polymeric microneedles.

Reference	Materials	Structure of microneedles	Shapes of microneedles	Dimensions	Array size/Needles	Analysis type	Testing	Fabrication techniques	Application
Park *et al*. 2010 [182]	PLA	Solid/Out-of- plane	Canonical/Square base	L = 600 μm W_b_ = 250 μm	10 × 10	Diffusion of trypan blue	Human/Porcine cadaver skin	UV lithography	Transdermal drug delivery
Gomaa *et al*. 2010 [183]	PMVE/MA	Solid/Out-of- plane	Canonical	L_1_ = 400 μm L_2_ = 600 μm L_3_ = 100 μm	11 × 11 14 × 14 19 × 19	Effect of Skin Permeability with microneedle density	Human skin	Laser micromachining	Drug delivery
Donnelly *et al*. 2010 [184]	Polymeric (Gantrez)	Solid/Out-of- plane	Canonical	Not reported	Not reported	Statistical	Porcine skin	Molding process	Intradermal delivery
Bodhale *et al*. 2010 [133]	PGA	Hollow/Out-of plane	Side opened/Sharp tip	L = 200 μm D_i_ = 30 μm D_o_ = 150 μm D_b_ = 300 μm	25 × 25	Structural/Fluidic	Not reported	Hot embossing/UV excimer laser(Proposed)	Drug delivery
Matteucci *et al*. 2009 [185]	PMMA	Hollow/Out-of plane	Rounded tip/Sharp tip	L = 500 to 1100 μm Bevel angle = 30° to 40°	10 arrays	Not reported	Not reported	DXRL	Not reported
Han *et al*. 2009 [186]	PLLA	Solid/Out-of plane	Sharp 3D Tip/Grooves-embedded shaft	L = 880 ± 20 μm, W_b_ = 710 ± 15 μm T = 145 ± 15 μm	Not reported	Protein transportation analysis	Mouse skin/Serum	Lithography/Ni electroplating/PDMS replication/Hot embossing	Intradermal immunization
Jin *et al*. 2009 [187]	PMMA/PC	Solid/In-plane	Quadrangular/Pyramidal	L = 200–1500 mm	Not reported	Drug transportation	Mouse skin and serum	DXRL/Hot embossing	Transdermal drug delivery
Oh *et al*. 2008 [156]	PC	Solid/Out-of plane	Sharp tip/Spear	L = 200–500 μm	Not reported	Hydrophilic molecules transportation	Mouse skin	Molding/Hot embossing	To improve skin permeability for hydrophilic molecules
Emam *et al.* 2008 [188]	SU-8	Out-of-plane/Hollow	Sharp tip	L = 500 μm, W_b_ = 100 μm	Not reported	Fluid analysis	Not reported	Deposition/Lithography/Etching	Treatment of hydrocephalus
Aoyagi *et al*. 2007[166]	PLA	Solid/Out-of plane	Straight/Harpoon shape/Sharp tip	L = 400 μm W_b_ = 90, 120, 150, 230 μm T = 115 μm Tip angle = 10°, 20°, 30°, 40°	Not reported	Structural	Artificial skin of silicone rubber	Etching/Injection molding	Drug delivery
Hsu *et al*. 2007 [189]	SU-8 2050	Out-of-plane	V-groove	L_1_ = 236 μm L_2_ = 350 μm	Not reported	Not reported	Not reported	Molding/KOH etching	Biomedicine technology

**Notations:** L = Length of needle, T = Thickness, W_b_ = Base width, D_o_ = Outer diameter, D_i_ = Inner diameter, D_b_ = Base diameter.

**Table 6:** Recent review of SiO_2_, glass, stainless-steel, and metallic microneedles.

Reference	Materials	Structure of Microneedles	Shapes of microneedles	Dimensions	Array size/Needles	Analysis Type	Testing	Fabrication Techniques	Applications
Kim *et al*. 2010 [190]	Stainless steel	Solid/In-plane	Spear/Sharp tip	L = 700 μm W_b_ = 160 μm T = 50 μm	5 microneedles	Drug transportation/Statistical analysis	Mouse skin	Infrared Laser	Vaccine delivery
Kato *et al*. 2010 [191]	SiO_2_	Hollow/Out- of-Plane	Circular Tip	L = 77 μm D_o_ = 5.5 μm D_i_ = 3.5 μm	Not reported	Structural (Panitration)	Gelatin	DRIE/Micromachining	Cellular function analysis
Ding *et al*. 2009 [172]	Stainless steel	Solid/Hollow/Out-of-Plane	Tangentially cut tip	L = 245, 300–900 μm D_2_ = 200 μm	4 × 4 9 × 9	Drug transportation/Statistical	Mouse skin	Surface micromachining/Etching	Dermal diphtheria/Influenza vaccination
Jiang *et al*. 2009 [167]	Glass	Hollow	Elliptical tip opening	L = 3–4 cm	Not reported	Histological/Microscopic image analysis	Human cadaver eyes	Micropipette pulling technique	Intrascleral delivery
Jin *et al*. 2009 [187]	Ni	Solid/In-plane	Triangular/Pyramidal	L= 200–1500 mm	Not reported	Drug transportation	Mouse skin and serum	DXRL/Hot embossing	Transdermal drug delivery
Hou *et al*. 2008 [192]	T_i_-alloy	Hollow/Out- of-Plane	Not reported	L = 120 μm	10 × 10	Fluidic analysis	Not reported	Not reported	Transdermal drug delivery
Kolli and Banga 2008 [193]	Maltose	Solid/In-plane	Tetrahedron/Sharp tip	L = 500 μm D_t_ = 6 μm	27 needle per array	Drug transportation	Mouse skin/Jacketed Franz diffusion cells	Micro-molding	Transdermal drug delivery
Verbaan *et al*. 2008 [194]	Metal	Solid/Hollow	Triangular tip/Tapered shaft	L = 245, 300 μm D = 200, 300 μm Beveled angle = 45° D_b_ = 250 μm	4 × 4 6 × 6 9 × 9	Waters HPLC System	Human skin	Surface micromachining/Etching	Transdermal drug delivery
Parker *et al*. 2007 [168]	Ti	Hollow/In- plane	Spare/Sharp tip	L = 500, 750, 1000 μm W_b_ = 100 μm Tip taper angle = 60°	10 needles	Fluidic/Structural analysis	Pressure Testing apparatus	Bulk micromachining/Multilayer lamination	Drug delivery
Shibata *et al*. 2007 [162]	SiO_2_	Hollow/Out-of- plane	Circler tip/Cylindrical	L = 77 μm, D_o_ = 5.5 μm D_i_ = 3.5 μm	Not reported	Structural analysis	Gelatin	Photolithography/DRIE	Cell surgery
Kim and Lee 2007 [195]	Metallic	Hollow/Out-of- plane	Tapered tip	L_1_ = 200 μm T = 10 μm L_2_ = 400 μm T = 20 μm Tapering angle < 5°	10 × 10	Fluidic analysis	Not reported	SU-8 based UV LIGA	Drug delivery/Body fluid sampling
Tsuchiya *et al*. 2005 [171]	Ti	Hollow/Out-of- plane	Cylindrical	L = 1 mm D_i_ = 25 μm D_o_ = 60 μm	Not reported	Fluidic analysis	Not reported	Sputter deposition	Blood extraction

**Notations:** L = Length of needle, T = Thickness, W_b_ = Base width, D_o_ = Outer diameter, D_i_ = Inner diameter, D_b_ = Base diameter, D_t_ = Tip diameter.
